# Molecular architecture of native fibronectin fibrils

**DOI:** 10.1038/ncomms8275

**Published:** 2015-06-04

**Authors:** Susanna Maria Früh, Ingmar Schoen, Jonas Ries, Viola Vogel

**Affiliations:** 1 Laboratory of Applied Mechanobiology, Department of Health Sciences and Technology, ETH Zurich, Vladimir-Prelog-Weg 4, Zurich 8093, Switzerland; 2 European Molecular Biology Laboratory, Cell Biology and Biophysics Unit, Meyerhofstrasse 1, Heidelberg 69117, Germany

## Abstract

Fibronectin fibrils within the extracellular matrix play central roles in physiological and pathological processes, yet many structural details about their hierarchical and molecular assembly remain unknown. Here we combine site-specific protein labelling with single-molecule localization by stepwise photobleaching or direct stochastic optical reconstruction microscopy (dSTORM), and determine the relative positions of various labelled sites within native matrix fibrils. Single end-labelled fibronectin molecules in fibrils display an average end-to-end distance of ∼133 nm. Sampling of site-specific antibody epitopes along the thinnest fibrils (protofibrils) shows periodic punctate label patterns with ∼95 nm repeats and alternating N- and C-terminal regions. These measurements suggest an antiparallel 30–40 nm overlap between N-termini, suggesting that the first five type I modules bind type III modules of the adjacent molecule. Thicker fibres show random bundling of protofibrils without a well-defined line-up. This super-resolution microscopy approach can be applied to other fibrillar protein assemblies of unknown structure.

## Abstract (web summary)

Fibronectin fibres are an important component of the extracellular matrix, supporting cell adhesion, growth and migration. Here the authors combine site-specific protein labelling with single-molecule localization microscopy to provide detailed insights into the molecular organization of native fibronectin fibrils.

## Graphical abstract

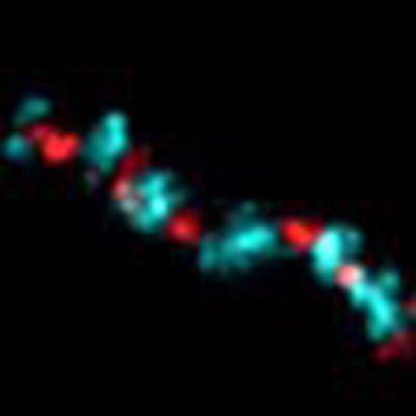

The adhesion protein fibronectin (Fn) forms an interconnected network of extracellular matrix (ECM) fibres that provides structural support and many biochemical cues to cells^1^,^2^. The organization of the Fn matrix plays a central role for the development and regeneration of tissues by coordinating cell adhesion, growth, migration and differentiation^3^,^4^,^5^. Moreover, Fn ECM is known to act as a template for the organization of other extracellular matrix proteins^6^. Abnormal expression levels of Fn in the ECM have also been associated with diseases such as fibrosis^7^ or cancer^8^,^9^. Thus, unravelling the native architecture of the Fn matrix is crucial to better understand how the structure and thus functional display of fibronectin fibres is related to physiological or pathological processes, and how cell-generated tension affects this relation^10^.

To elucidate the relationship between Fn conformation and function, the relative arrangement and interactions of Fn molecules in fibrils needs to be known. Current models for the architecture of Fn fibrils are based on structural as well as functional data^11^,^12^. Crystal and solution structures of short recombinant Fn constructs revealed the fold and dimensions of type I, II and III modules^13^,^14^,^15^. Electron microscopy of individual Fn molecules adsorbed to surfaces showed an extended and flexible conformation of Fn dimers with a variable contour length of 120–160 nm (ref. 16), implying a beads-on-a-string arrangement of modules. Direct or competitive protein binding studies and matrix incorporation assays using proteolytic or recombinant Fn fragments or antibodies showed that Fn dimerization via the C-terminal disulfide bridges^17^, the N-terminal type I modules FnI_1-5_ (refs 17, 18, 19, 20, 21), and at least one of the two first type III modules FnIII_1-2_ (refs 21, 22, 23, 24, 25) are essential for fibril formation. Electron microscopy images of Fn fibrils produced in cell culture showed fibrils of distinct (smooth or nodular) morphology with thicknesses ranging from 5 to 20 nm, but could not reveal the arrangement of single molecules within these fibrils^26^,^27^. Immunogold labelling of extra domain A in cellular Fn matrix revealed a regular arrangement with prevailing distances of 70–110 nm along fibrils^28^.

On the basis of all these findings, a consensus has been reached that Fn dimers polymerize in an end-to-end manner and that the essential interactions between adjacent molecules are mediated by an N-terminal overlap. However, it is controversial whether FnIII_1-2_ (ref. 29) and even FnIII_4-5_ (ref. 30) are part of the N-terminal overlap or merely mediate lateral bundling of protofibrils^31^. Moreover, a recent study has shown that the domain FnI_6_-FnI_9_ (comprising the gelatin binding domain, GBD) forms a compact structure with dimerization capacity^32^,^33^, but the physiological implications of these findings remain unclear. Finally, the structure of bundled fibrils is elusive.

Single-molecule localization microscopy (SMLM) is a novel and powerful set of tools for structural biology^34^. SMLM comprises a number of related ‘super-resolution' techniques like photoactivated localization microscopy (PALM)^35^, stochastic optical reconstruction microscopy (STORM)^36^, direct stochastic optical reconstruction microscopy (dSTORM)^37^ or stepwise photobleaching^38^,^39^. These utilize different mechanisms to switch the fluorescence of labels on and off over time and sequentially measure the position of single fluorophores. When combined with site- and/or molecule-specific labelling strategies, SMLM can reveal structural details of protein complexes with sub-molecular resolution^40^,^41^. A recent STORM study, for example, showed for the first time periodic actin/spectrin structures along the axon of neurons^42^. In contrast to these structures that were composed of tens to hundreds of molecules, the resolution of complexes that contain only a small number of molecules remains challenging. In particular, incomplete and inaccurate labelling, as well as the stochastic nature of most SMLM techniques itself constitute major hurdles for quantitative analyses and call for customized strategies, such as single particle averaging^43^,^44^.

In this study, SMLM is used to investigate the hierarchical and molecular architecture of Fn matrix fibrils assembled by normal human dermal fibroblasts (NHDFs). We employ matrix incorporation of fluorescent Fn conjugates or labelling of distinct epitopes by immunofluorescence and developed quantitative analysis procedures to determine the relative arrangement and distances between individual labelled sites. The measurements reveal the basic periodicity of fibrils, the arrangement and extension of Fn molecules therein, as well as insights into fibril bundling.

## Results

**Figure 1:**
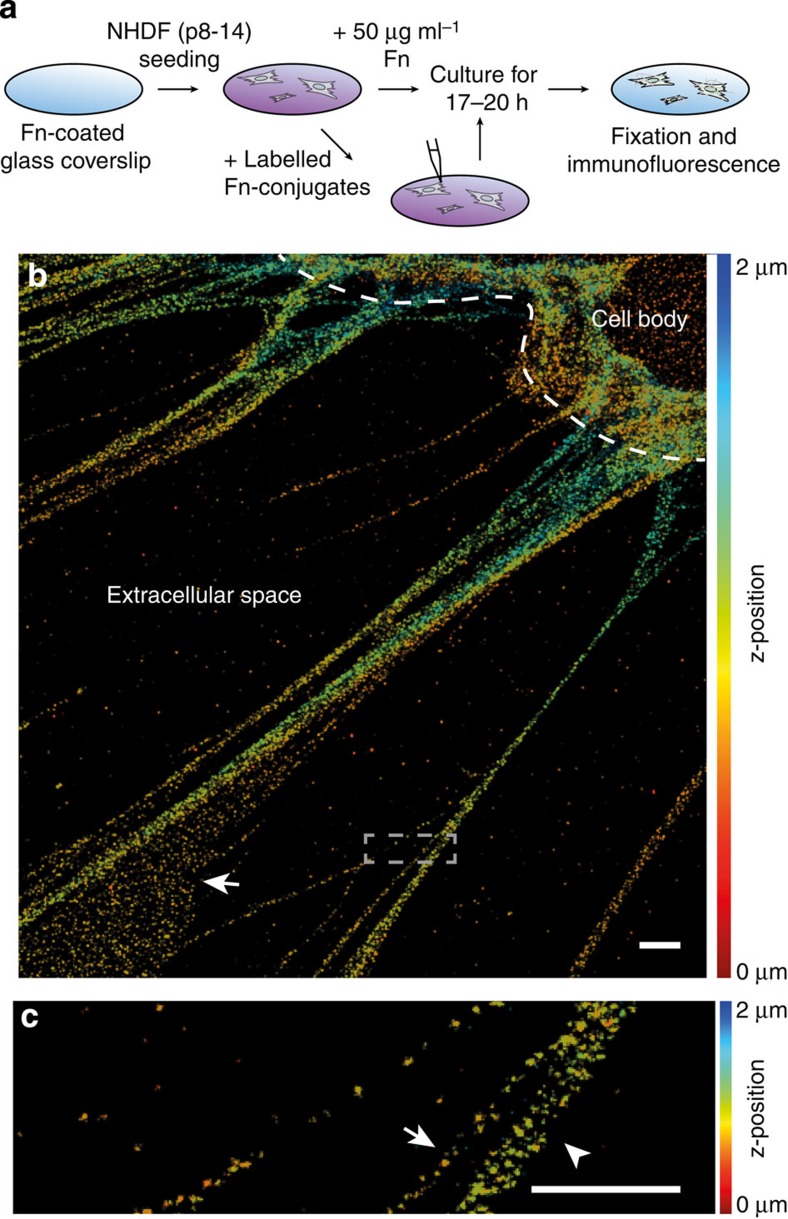
Fibronectin extracellular matrix assembled by fibroblasts in cell culture and imaged by direct stochastic optical reconstruction microscopy (dSTORM). (**a**) Experimental design and Fn-labelling steps. Normal human dermal fibroblasts (NHDFs, passage 8–14) were cultured for 17–20 h on a Fn-coated glass surface in a Fn-enriched medium. (**b**) Gaussian rendering of a 3D dSTORM image of Fn matrix fibrils assembled by NHDFs and immunolabelled with the IST2 antibody. The dashed white line depicts the cell edge. Arrow: area with surface adsorbed Fn molecules. The z-position was colour-coded from red (towards the coverslip) to blue (towards the apical cell surface). Scale bar, 1 μm. (**c**) Magnification of the boxed region in (**b**) showing punctate epitope patterns sampled by the IST2 antibody. Examples for a thin fibril (white arrow) and a thick fibril (arrowhead) are indicated. Scale bar, 1 μm.

To investigate the hierarchical structure of Fn fibrils within native ECM by SMLM, NHDFs were cultured on Fn-coated glass coverslips for 17–20 h in medium supplemented with 50 μg ml^−1^ plasma Fn (pFn) (Fig. 1a). The cell-assembled Fn matrix was mainly composed of pFn due to the short culture duration and the excess of pFn in the medium (Supplementary Fig. 1). Two labelling approaches were exploited here. Fixed samples were stained by immunofluorescence, mounted and imaged. Alternatively, 0.1–5% of exogenous pFn was fluorescently labelled, added to the medium and incorporated by cells into matrix fibrils. Figure 1b shows a 3D dSTORM image of Fn matrix labelled with an anti-Fn antibody (IST2). All dSTORM images are shown in a Gaussian rendering of the localizations. Peripheral Fn fibrils emanated from the coverslip, interconnected, exhibited different thicknesses and were oriented towards the cell. For characterizing the structure of Fn fibrils, we focused our analysis on the interconnected 3D network in the extracellular space and selected those Fn fibrils for further analysis where a clear identification of single fibrils or of fibril-fibril junctions was possible. Thus, simply surface adsorbed labelled molecules were excluded from further analysis (white arrow in Fig. 1b). The subset of analysed filaments exhibited apparent thicknesses of 20 nm, which is the lower limit given by the localization imprecision, up to more than 100 nm (Supplementary Fig. 2). To study the molecular architecture of Fn fibrils, we first analysed the thinnest fibrils in the extracellular space. We find that these thinnest Fn fibrils are characterized by a punctate appearance of labelled epitopes (Fig. 1c, white arrow), and will from here on call these thinnest Fn fibrils ‘protofibrils'.

### Extension of single Fn molecules within protofibrils

**Figure 2:**
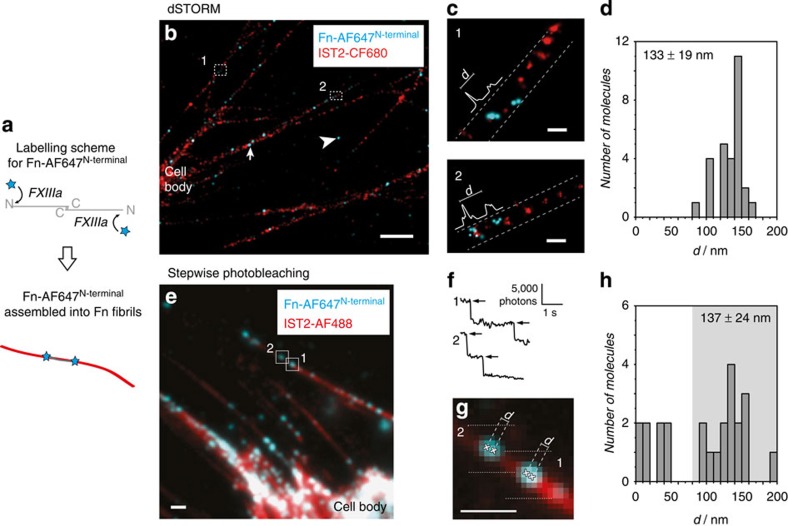
Extension of single N-terminally labelled Fn dimers in the thinnest Fn fibrils (protofibrils). (**a**) A specific labelling of Fn N-termini was achieved by activated blood coagulation factor XIII (FXIIIa)-catalysed transamidation. Pre-labelled Fn-AF647^N-terminal^ conjugates were incorporated into the Fn ECM at high dilution for measuring the end-to-end distances of single molecules. (**b**) Dual colour dSTORM with Fn-AF647^N-terminal^ (blue) and IST2-CF680 (red). The blue channel is represented in an overexposed way for better visualization of the sparse Fn-AF647^N-terminal^ labels. Arrowhead: surface-adsorbed molecules. Arrow: signals in a thick fibril where no clear identification of the protofibril was possible. Both cases did not enter the analysis. Scale bar, 1 μm. (**c**) Magnification of areas (1 and 2) from (**b**) and schematic measurement of the inter-label distance in the blue channel. Scale bar, 100 nm. (**d**) Histogram of measured inter-label distances (*n*=28, from 11 independent cells) from dSTORM measurements. Numbers are given as mean +/− s.d. (**e**) First frame from a stepwise bleaching movie with Fn-AF647^N-terminal^ (blue) overlayed with an epi-fluorescence image of the IST2-AF488 stain (red). The image contrast has been adjusted for better visualization. Scale bar, 1 μm. (**f**) Examples of intensity time traces of individual Fn-AF647^N-terminal^ molecules (1 and 2) from (**e**) that were bleached in two steps and assigned to a fibril via the reference colour image. Arrows indicate bleaching events of single fluorophores. Scale bar: photons per frame versus time. (**g**) Magnification from image (**e**) with the two molecules 1 and 2. Crosses: fluorophore positions and respective distances as determined from the stepwise photobleaching movie. Scale bar, 1 μm. (**h**) Histogram of measured inter-label distances (*n*=24, from 18 independent cells) by stepwise photobleaching. The mean +/− s.d. of distances >80 nm (grey area, *n*=16) is given. The origin of smaller distances is discussed in the main text.

While the contour length of single surface-adsorbed Fn molecules has been measured^16^, their extension in the fibrillar environment is not known. Instead of sampling the contour of individual lysine-labelled Fn molecules in matrix fibrils that is complicated by the random distribution of labels (Supplementary Fig. 3), we measured the end-to-end distance of single Fn dimers that were site-specifically labelled at their N-termini using an established protocol^45^. The fluorescent conjugates Fn-AF647^N-terminal^ were added during cell culture at very dilute concentrations to ensure that the density of incorporated molecules in Fn fibrils was low enough to identify single molecules (Fig. 2a). To visualize the context of isolated Fn molecules, the Fn matrix was further immunolabelled after fixation using the IST2 antibody and a separate fluorophore colour.

Dual colour dSTORM images showed the characteristic punctate staining of Fn protofibrils in the IST2-CF680 channel and only few isolated fluorescence signals of N-terminally AF647-labelled Fn (Fig. 2b). Bright signals that were not part of the Fn matrix (arrowhead) corresponded to adsorbed, pre-labelled Fn molecules at the coverslip and were excluded from further analysis. Very bright signals in thicker fibres (arrows) where no clear identification of the corresponding protofibril was possible were excluded as well. A closer analysis of the isolated AF647 signal in single protofibrils revealed two distinct spots (Fig. 2c). Each pair of spots was oriented along the Fn fibril indicating that the Fn dimers were aligned with the fibre axis. The distance between the paired spots was determined by fitting its line profile with the sum of two Gaussians and taking the distance between peak positions. The measured distances ranged from 90 to 160 nm, with a mean and s.d. of 133±19 nm (Fig. 2d, *n*=28). The width of the measured end-to-end distance distribution of Fn dimers exceeded the expected error according to the localization precision in our dSTORM measurements and thus also comprised a natural variability between different Fn dimers in these fibrils.

To validate that a pair of spots belonged to a single Fn dimer and not to two different Fn molecules, we also measured the extension of the N-terminally labelled Fn dimers by stepwise photobleaching that directly counts the number of fluorophores per diffraction-limited spot. Spots with many bleaching steps represented aggregated or near-by molecules and were excluded from the analysis. To ensure that we were measuring single molecules, only spots with two bleaching steps were used for the localization. In addition, an epifluorescence image of the Fn matrix in the separate IST2–488 channel was used to visualize the Fn matrix and identify isolated thin fibrils (Fig. 2e). During the acquisition of movies in the AF647 channel, the intensity of fluorescent spots decreased in distinct steps that correspond to bleaching events of individual fluorophores (Fig. 2f) and resembled the known bleaching behaviour of Fn-AF647^N-terminal^ (ref. 45). Like in dSTORM images, the fitted positions of the N-terminal labelling sites of Fn dimers were aligned along the direction of the protofibril (Fig. 2g). A histogram of the measured distances (Fig. 2h) showed two populations, a smaller one with distances between 4 and 40 nm (*n*=8), and a larger one with distances between 90 and 190 nm (*n*=16). The smaller fraction presumably corresponded to molecules that carried two fluorophores at the same N-terminus (which arise as a side-product of the labelling procedure^45^), or to compact, adsorbed molecules. The dominant population at larger distances (Fig. 2h, grey area) had a mean and s.d. of 137±24 nm and resembled the distance distribution obtained by dSTORM (Fig. 2d). We thus conclude that both methods for measuring the extension of Fn dimers within fibrils yielded consistent results.

### Periodicity of selected epitopes within Fn protofibrils

**Figure 3:**
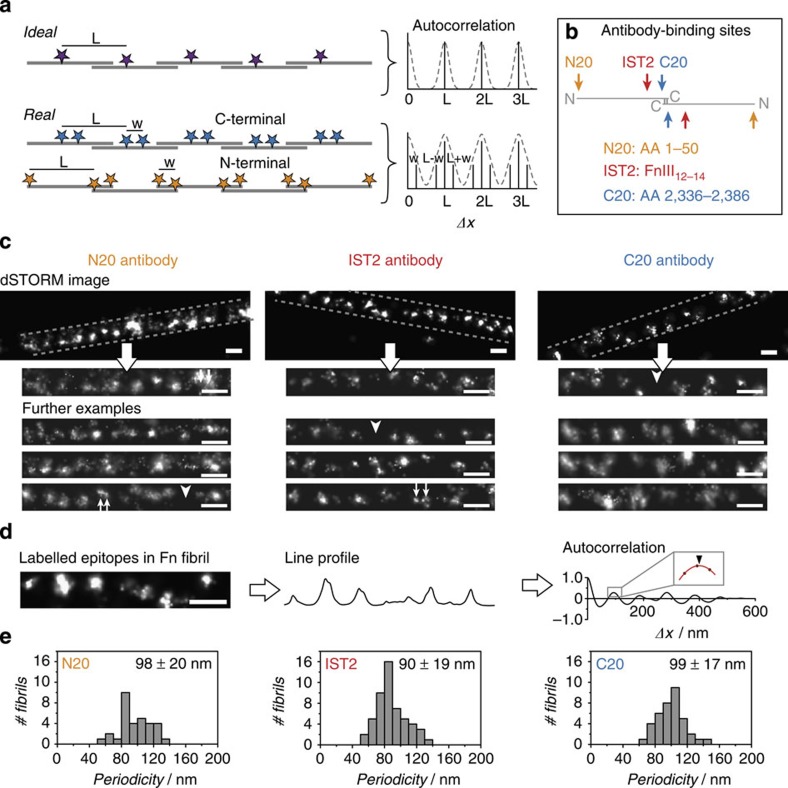
Periodicity of labelling patterns in the thinnest Fn fibrils (protofibrils). (**a**) Principle for determining Fn fibril periodicity. Top: ideal case in which only one site is labelled in each Fn molecule. Bottom: real case in which each Fn dimer contains two epitopes for the same antibody. Right: autocorrelation of the label patterns for infinite (solid black lines) and finite (dashed grey lines) localization precision. (**b**) Location of Fn binding sites for antibodies N20 (yellow), IST2 (red) and C20 (blue). The amino acid (AA) region and the targeted modules are indicated below. (**c**) Sampling of epitopes by immunolabelling using antibodies N20 (left), IST2 (middle) or C20 (right). Top: overview of labelled epitopes in the Fn fibril and selection of regions for further analysis. Dashed lines frame the Fn fibril. Bottom: three further examples for dSTORM images of fibrils sections for each antibody. Arrowheads: void labelling sites. Small arrows: double peaks. (**d**) Workflow of the periodicity analysis: an intensity line profile along a fibril section was obtained from a dSTORM image (here: C20 antibody) and the first peak in its autocorrelation was taken as a measure of fibril periodicity. Inset: refinement of the peak position (arrowhead) by a quadratic interpolation (red line). (**e**) Histograms for the periodicity of N20, IST2 and C20 stainings (*n*=32/53/39 from 12/18/14 independent cells, respectively). Numbers are given as mean +/− s.d. All scale bars 100 nm.

To obtain positional information regarding the relative arrangement of Fn dimers with respect to each other, specific epitopes on Fn were immunolabelled such as to maximize the labelling efficiency of accessible epitopes. Since Fn dimers were found to be aligned along the fibre axis (Fig. 2c,g), their linear, overlapping arrangement should repeat itself along a Fn protofibril. This repeat length (‘L') could be directly inferred from the distances between labels if a single site in each Fn dimer was labelled (Fig. 3a, top). However, site-specific immunolabelling yields two labelled sites per Fn dimer (one on each monomer) (Fig. 3a, bottom), which deteriorates the analysis of inter-label distances. An autocorrelation analysis can overcome this problem and reveal the periodicity along a protofibril, as long as the spacing between nearby labelling sites in Fn protofibrils is much smaller than the characteristic repeat length (Fig. 3a, right). To comply with this condition, we sampled different epitopes situated close to the N- or C-termini of Fn (Fig. 3b). Antibodies N20 and C20 raised against sequences at the respective termini, as well as the IST2 antibody that recognizes a site close to FnIII_12-14_, were used. dSTORM images of the thinnest fibrils revealed distinct punctate patterns along the fibril contour with an apparent regularity on the length scale of roughly 100 nm (Fig. 3c). As higher antibody concentrations (antibody dilution series from 1:200 to 1:25) did not change the punctate appearance, our finding does not represent an under-sampling due to incomplete labelling but rather reflects the fact that antibody epitopes were concentrated at distinct regions along protofibrils.

To quantify the periodicity of single epitopes along a fibre, we analysed their spatial autocorrelation. This analysis considers and partially compensates for stochastic variations that are present in the dSTORM images (see Supplementary Note): the scattering (‘inaccuracy') of label positions (Fig. 3c) as expected from an arbitrary orientation of primary/secondary antibodies, voids in otherwise regular patterns (Fig. 3c, arrowheads) due to unlabelled sites, or split peaks (Fig. 3c, arrows) arising from slightly different positions of epitopes (as explained in Fig. 3a). From each dSTORM image, an intensity line profile along the fibril was generated and correlated with itself^42^. If the autocorrelation showed regularly spaced maxima up to fourth or higher order (Fig. 3d), fibrils were considered to be periodic. The position of the first peak in the autocorrelation was then refined by a quadratic interpolation and taken as a quantitative measure of fibril periodicity. This procedure was tested against stochastic simulations and yielded robust and accurate results over a large range of localization parameters (see Supplementary Note). When applying this autocorrelation analysis to the images of Fn protofibrils, ∼65% of labelling patterns were identified as periodic. Non-periodic patterns may be attributed to either too large stochastic variations that compromise the periodicity (see Supplementary Fig. 4a), or to an irregular fibril structure composed of molecules with substantially different conformations. The measured periodicities ranged from ∼60 to 130 nm, and similar histograms were obtained with the different antibodies (Fig. 3e). The average periodicity and the s.d. were 98±20 nm (*n*=32) for the N20 stain, 90±19 nm (*n*=53) for IST2, and 99±17 nm (*n*=39) for C20. The difference between the distributions for the different antibodies was statistically not significant *(P*>0.01, One-Way ANOVA with Tukey-Test). The variation of fibril periodicity was larger than the precision of the autocorrelation analysis (see Supplementary Fig. 5b,c) and thus also comprises a natural variability between fibrils. In a negative control using randomly labelled microtubules, less line profiles were classified as periodic and yielded a wide distribution without distinct peak (Supplementary Fig. 6). We thus conclude that the observed label patterns in protofibrils originated from a regular structure with an average repeating unit of ∼95 nm in length.

### Sequential order of different epitopes along Fn protofibrils

**Figure 4:**
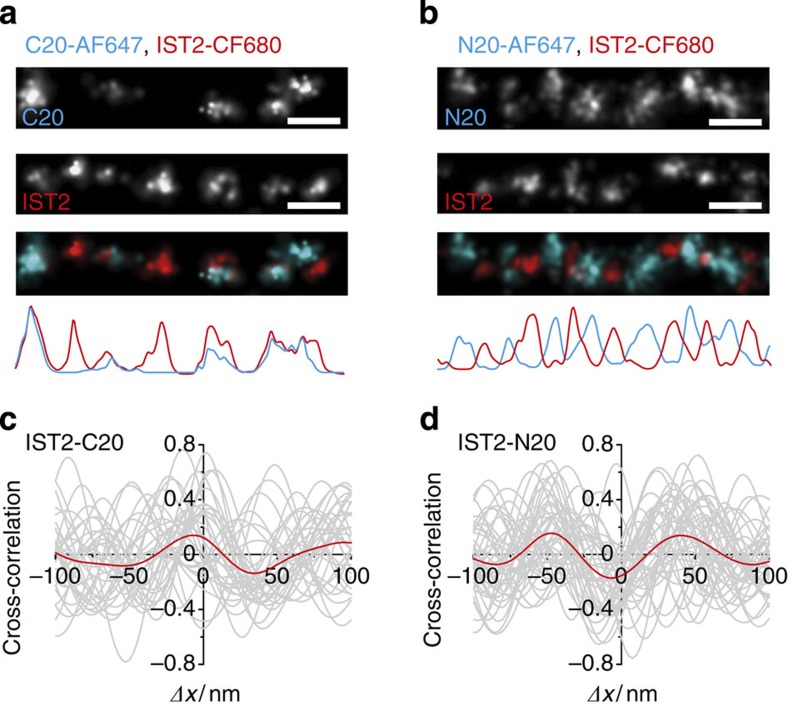
Spatial correlation between labelling patterns of different antibody epitopes along single Fn protofibrils. (**a**) Dual colour immunolabelling of Fn protofibrils with C20-AF647 and IST2-CF680. Top: dSTORM images of an exemplary fibril section and a red-blue overlay. Bottom: line profiles. (**b**) Dual colour immunolabelling of Fn protofibrils with N20-AF647 and IST2-CF680. (**c**) Cross-correlation between IST2 and C20 labelling patterns (*n*=34, grey, from seven independent cells). Red line represents the mean. (**d**) Cross-correlation between IST2 and N20 labelling patterns (*n*=44, grey, from eight independent cells). All scale bars 100 nm.

To study the relative positions of different epitopes along thin Fn fibrils, we next used dual colour dSTORM. Regular label patterns were observed in both channels for the combination of C20 and IST2 antibodies (Fig. 4a), as well as for N20 and IST2 (Fig. 4b). Image overlays and line profiles (Fig. 4a,b, bottom) showed that C20 tended to co-localize with IST2 antibodies, whereas positions of N20 were shifted relative to IST2 (see also Supplementary Fig. 5a). A cross-correlation analysis between the two different stains quantitatively confirmed this finding: the averaged cross-correlation was in-phase for C20-IST2 (Fig. 4c, *n*=34) and anti-correlated for N20-IST2 (Fig. 4d, *n*=44). In both cases, the periodicity agreed with that obtained from the previous autocorrelation analysis. These results showed that N- and C-terminal regions were alternating along Fn protofibrils.

### Characterization of fibril bundling in thicker Fn fibres

We finally addressed the hypothesis that thicker fibres were formed from thinner fibrils by bundling^12^,^28^. To this end, we characterized the joining of fibrils at fibril junctions, as well as the periodicity of antibody labelling patterns in thick fibrils.

**Figure 5:**
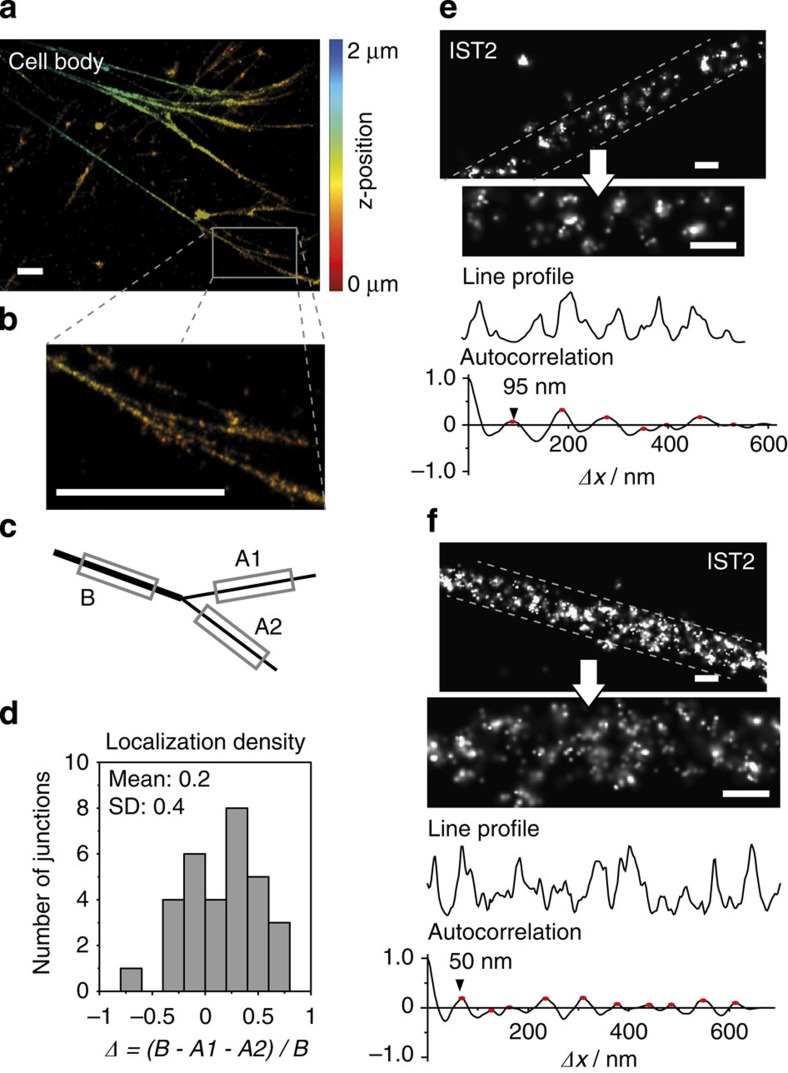
Characterization of protofibril bundling into thicker Fn fibres. (**a**) 3D dSTORM image of Fn matrix in the extracellular space. The matrix was stained by incorporation of lysine-labelled Fn molecules. Scale bar, 1 μm. (**b**) Magnification of the boxed region in (**a**) showing the joining of two fibrils. Scale bar, 1 μm. (**c**) Schematic for the analysis of fibril segments before (*B*) and after (*A1*, *A2*) a junction. (**d**) Histogram of relative differences in the localization densities before and after a junction (*n*=31, from five independent cells). (**e**,**f**) Example dSTORM images of thick fibres and selection of regions for further analysis (top) and their respective line profile (middle) and autocorrelation (bottom). The fibre in (**e**) showed a periodicity of ∼95 nm, the fibre in (**f**) had a periodicity <60 nm. Scale bars: 100 nm.

Fibrils that emanated from junctions towards to the cell appeared brighter and thicker than those towards distal locations (Fig. 5a,b). As a quantitative measure for the amount of Fn per fibril, we determined the localization density (number of localized fluorophores per length) along fibril sections. The localization density difference between the fibril before and the sum of fibrils after a junction was normalized to the fibril before the junction (Fig. 5c). This relative difference (*Δ*) was used as a thickness-independent measure for fibril bundling: *Δ*=0 represents pure bundling, whereas *Δ*>0 (*Δ*<0) requires a gain (loss) of Fn molecules upon bundling. The obtained distribution (Fig. 5d) had a width of approximately±30% and its mean value was not significantly different from zero (*P*>0.01, one-sample *t*-test). Alternatively, an analogue analysis of fibril thicknesses before and after junctions did neither show a significant difference (Supplementary Fig. 7a; *P*>0.01, one-sample *t*-test). Both results support the view that thicker fibres are the sum of thinner fibrils.

We thus asked whether the thick fibrils that consist of bundled protofibrils would also show the periodicity of protofibrils. An IST2 antibody staining along thick fibrils revealed periodic sections (Fig. 5e,f) in 50% of analysed patterns. The periodicity was comparable to that of thin fibrils in some cases, while in others it was distinctly shorter, on the order of 60 nm (see also Supplementary Fig. 7c). As an internal test, we analysed dual colour stainings of single protofibrils as if they were representing two bundled fibrils (Supplementary Fig. 5d–g). We indeed obtained similar periodicities: larger ones for C20-IST2 (Supplementary Fig. 5f), and shorter ones for N20-IST2 (Supplementary Fig. 5g). The observed periodicities in thick fibrils thus are consistent with parallel protofibrils that had different shifts with respect to each other. Together, these experiments showed that the Fn matrix is based on a hierarchical assembly of protofibrils.

## Discussion

Gaining insights into the molecular arrangement in Fn fibrils within the ECM has been a long-standing dream^31^,^46^, which we addressed here by taking advantage of recent developments in sub-diffraction fluorescence microscopy methods^34^. The use of site-specific labelling techniques and SMLM enabled us to conduct for the first time a quantitative investigation of the architecture of thin Fn fibrils in cell culture. Our results provide structural information that complements previous studies using electron microscopy, protein interaction or matrix incorporation assays^31^, and taken together yield a comprehensive picture of the hierarchically assembled structure of Fn fibrils.

Labelling of the two N-termini in Fn dimers revealed an average end-to-end distance of ∼133 nm and an aligned orientation along the fibril axis (Fig. 2). The range of measured end-to-end distances quantitatively agreed with the range in contour length of individual, surface-adsorbed Fn molecules in electron microscopy images^16^, and the maximum extension of ∼190 nm corresponds to a straightened-out Fn dimer assuming a linear arrangement of intact modules (∼180–190 nm). Hence, dimeric Fn molecules in thin fibrils adopted an extended yet, in most cases, not fully stretched conformation. This finding is also in agreement with conclusions from fluorescence resonance energy transfer (FRET) measurements of Fn conformation in the ECM^47^.

Even though high-resolution electron microscopy of native Fn fibrils do not show an apparent periodic substructure^26^,^27^, we discover here that most Fn dimers within the thinnest fibrils do align in a periodic manner. Different N- and C-terminal epitopes along thin Fn fibrils each had a dominant periodicity ranging from 60 to 130 nm, with ∼95 nm on average, as revealed by our autocorrelation analysis (Fig. 3e). This approximately matches the ∼84 nm distances obtained by a nearest-neighbour analysis from electron microscopy images using immunogold labelling of extra domain A^28^. Potential differences between cellular versus plasma fibronectin matrices are expected to be small (as the insertion of the extra domains A and B increases the length of a Fn dimer by only ∼5%), and could not be detected in a control experiment (Supplementary Fig. 8). Importantly, only the autocorrelation analysis can distinguish a periodic from a random arrangement of labels (see Supplementary Fig. 6), whereas the distribution of nearest-neighbour distances between labels always peaks at the most prominent distance, even for a purely random arrangement. Our findings thus unambiguously demonstrate for the first time that the Fn protofibrils are periodic structures. Solely in longer (>1 μm) fibril sections, the periodic arrangement was eventually lost, presumably due to local defects in fibril composition or due to substantial fibril strain^47^,^48^,^49^.

**Figure 6:**
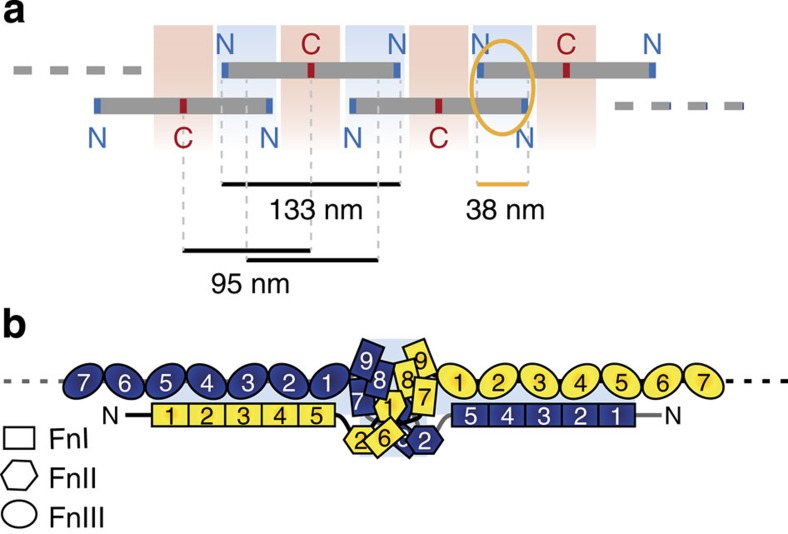
Model for the arrangement and interaction of adjacent Fn molecules in protofibrils. (**a**) The measured length of single Fn molecules (see Fig. 2), the periodicity of labels (see Fig. 3) and the alternating C- and N-terminal regions (see Fig. 4) suggest a staggered, linear arrangement with substantial overlap between adjacent Fn molecules. Fn dimers are depicted as grey sticks, their N-termini are marked in blue, C-termini are represented in red. (**b**) Proposed model for the N-terminal overlap. The following inter-molecular interactions could stabilize the proposed configuration: FnI_1-5_ interacting with FnIII_1-5_, dimerization of GBD (FnI_6_ to FnI_9_), and FnIII_1-5_ interacting with FnI_1-5_ from the antiparallel Fn molecule. As the structure of the GBD domain within fibrillar Fn is not known, our sketch was guided by the only available GBD dimer structure, even though it was obtained in the presence of Zn^2+^ (ref. 32). Similarly, no structure has been reported so far for the 16-amino-acid-long linker between FnI_5_ and FnI_6_.

We further find that the formation of Fn fibrils is mediated by a substantial N-terminal overlap. The alternating arrangement of N- and C-terminal regions along thinnest Fn fibrils (Fig. 4) requires an aligned, staggered arrangement of Fn dimers. The overlap between adjacent molecules can be deduced by subtracting the length of the repeating unit, which is given by the fibril periodicity (Fig. 3), from the length of an extended molecule, which is given by its end-to-end distance (Fig. 2). The average measured epitope periodicity of 95±2 nm and the average end-to-end distance of 133±4 nm from dSTORM measurements imply an overlap of ∼38±5 nm (Fig. 6a), which is distinctly longer than previously thought^28^. It is important to note that the conclusion about the periodic arrangement of molecules in protofibrils remains unchanged even if the investigated fibrils were more than one molecule thick. A major offset between parallel molecules in protofibrils, however, would not be consistent with the observed alternation of N/C-terminal sequences.

The measured N-terminal overlap is distinctly larger than what would be expected from a linear arrangement of the first five type I modules (FnI_1_-FnI_5_; 5 × 2.8 nm (ref. 13) ∼14 nm). Our measurements hence are not compatible with models in which fibril formation is based on the anti-parallel binding of FnI_1-5_ regions of adjacent molecules alone^17^,^28^. Instead an overlap of 30–40 nm supports recent models^29^,^50^ in which FnIII_1_ and/or FnIII_2_ of one molecule interact with parts of the FnI_1-5_ sequence of the adjacent molecule (Fig. 6b). Such a configuration also allows for additional interactions between FnIII_1-5_ and FnI_1-5_. It is thus in agreement with findings that FnIII_4-5_ (ref. 30) and the presence of all five type I modules FnI_1-5_ (ref. 17) are essential for fibril formation, as well as with the multiple interaction possibilities that were described for individual FnIII_1_ or FnIII_2_ domains along FnI_2-5_ (ref. 50). This arrangement further allows interactions between the GBD (FnI_6_-FnI_9_) regions of neighbouring molecules. GBD homodimer formation was observed in the presence of divalent metal ions and yielded a crystal structure with a tertiary fold of approximately 4 × 4 × 8 nm (ref. 32). In agreement with these findings, the measured overlap suggests that the dimension of the GBD is less than ∼7 nm, which is distinctly shorter than the linear arrangement of four FnI and two FnII modules (4 × 2.8 nm+2 × 2.5 nm=13.8 nm).

Thicker as well as more mature Fn fibrils consist of random bundles of protofibrils (Fig. 5). The bundling of Fn fibres in cell culture was observed long ago^51^, but the details remained unclear. Here we see that protofibrils are arranged side by side with a certain shift (Fig. 5e,f); however, this relative shift varied from fibre to fibre (Fig. 5e,f). Our results thus suggest that the staggering between bundled protofibrils is rather random, in contrast to previous models that assumed distinct shifts^28^. It thus seems unlikely that protofibril–protofibril interactions involve only a small number of specific sites (including FnIII_1-2_ and FnIII_4-5_ (ref. 31)); instead, several additional interactions along the Fn fibril could further stabilize protofibril bundling, including electrostatic interactions between oppositely charged regions^52^ or β–strand exchange between FnIII modules^53^, as previously suggested.

Our structural findings also shed new light on the structural basis of the exceptional mechanical properties of Fn fibrils. On the protofibril level, the large N-terminal overlap (Fig. 6b) yields an extended interface between interacting molecules. In this overlap region, many parallel Fn-Fn bonds can form a multivalent cluster of bonds, which have to be broken all at once in a single rupture event when the fibril is stretched. This ‘shear'-geometric arrangement of noncovalent connections is the hallmark of mechanically robust intra- and inter-molecular bonds in nature^54^,^55^. The large binding interface thus could potentially explain the high mechanical stability of bundled native Fn fibrils^48^ and presumably also of artificial Fn fibrils^56^ as well as their insolubility to detergents^57^. On the level of thicker fibres, the bundling of protofibrils enhances mechanical stability by distribution of stress among parallel fibrils. The relative shifts between protofibrils might affect the sequence in which Fn repeats that differ in mechanical stability unfold upon fibre stretching, as well as the refolding kinetics^58^. In this regard, the suggested random bundling could be advantageous to maintain fibril integrity over many stretch-relax cycles or under excessive stress.

The combination of super-resolution microscopy with site-specific labelling opens the door to further explore how the nanoscale spatial arrangement of binding sites along a Fn fibril for integrins^59^ and cofactors^5^, as well as physical properties of the substrate^60^ induce membrane receptor clustering and eventually trigger cell signalling. Finally, the tertiary structure of the N-terminal overlap region has implications for the exposure and mechanical stability of local binding sites for bacterial Fn binding peptides (FnBPs), collagen, as well as for gelatin and heparin^10^. For some of these, it was already shown that tensile forces could regulate ligand affinity in a strain-dependent manner^61^,^62^.

## Methods

### Purification and site-specific labelling of Fn

Human plasma (Blutspende Zurich, Schlieren, Switzerland) was passed through a size-exclusion column (#17-0851-01, GE Healthcare) and loaded onto a gelatin Sepharose 4B column (#17-0956-01, GE Healthcare). After washing with phosphate buffered saline (PBS), NaCl (1 M in PBS) and arginine (0.2 M in PBS), Fn was eluted with 1 M arginine (in PBS). Fn purity was checked by SDS-PAGE and western blotting. The purified Fn (in 1 M arginine in PBS) was aliquoted and stored at −80 °C.

A random labelling of surface accessible lysine residues of Fn was obtained by amid bond formation with fluorescent probes. Fn was transferred into an amine labelling buffer (PBS with 0.1 M NaHCO_3_, pH 8.5) by size-exclusion chromatography (Sephadex PD-10 column, GE Healthcare). The elutant was incubated with a 150-fold molar excess of Alexa Fluor 647 (AF647) succinimidyl ester (#A20006, Invitrogen) for 1 h at room temperature under exclusion of light. Free dye was removed and the buffer exchanged to PBS using a Sephadex PD-10 column. The Fn-AF647 batch used in this study carried 22 dyes per molecule on average as measured by absorption.

Site-specific labelling of Fn at its N-terminal tails was achieved by activated blood coagulation factor XIII (FXIIIa)-mediated transamidation with a fluorescently labelled peptide Ac-FKGGGC(Alexa Fluor 647)-NH_2_ as described elsewhere^45^. The Fn-AF647^N-terminal^ batch used in this study carried 1.9 dyes per molecule on average, with about ∼64% of molecules labelled with 1 dye at each tail (see^45^ for a detailed characterization). Fluorescent Fn conjugates were aliquoted and stored at −20 °C in the dark.

### Cell culture

Coverslips (18 mm diameter; thickness 1.5, Hecht-Assistent) were coated with Fn (50 μg ml^−1^ in PBS) for 1 h, washed three times with PBS and sterilized under UV for 1 h. Normal human dermal fibroblasts (NHDF, passage 8–14; #c-12,300, PromoCell) were seeded onto the glass coverslips at a density of 4,500 cells cm^−2^. Cells were cultured for 17–20 h in MEM Alpha (#L0475–500, Biowest) supplemented with 10% fetal bovine serum (#S1810, Biowest). After 1 h pFn (50 μg ml^−1^ in medium) was added. For measuring end-to-end distances, 0.1–1% of the added Fn was replaced by Fn-AF647^N-terminal^. For measuring the contour length of lysine-labelled Fn, 0.1–1% of the added Fn was replaced by Fn-AF647.

### Sample fixation and fluorescence labelling

Samples were washed three times with PBS, fixed in formaldehyde (4% v/v in PBS) for 10 min and washed three times with PBS. For immunofluorescence labelling, fixed samples were incubated with primary antibodies in blocking buffer (3% w/v bovine serum albumin (#A6588, AppliChem) in PBS) for 1 h at room temperature. The samples were washed three times and stained with secondary antibodies in blocking buffer for 30 min at room temperature in the dark. For dual-colour labelling with secondary antibodies that had a cross-reactivity, antibodies were applied sequentially. Samples were briefly washed with PBS and then post-fixed with 4% v/v formaldehyde in PBS for 10 min, washed three times with PBS and stored at 4 °C in the dark.

### Antibodies

A rabbit polyclonal anti-Fn (20 μg ml^−1^, ab23750, Abcam) was used for the junction analysis. For sampling of N- or C-terminal epitopes along Fn fibrils, we used goat polyclonal anti-Fn N20 (1:50, #sc-6953, Santa Cruz) and goat polyclonal anti-Fn C20 (1:50, #sc-6952, Santa Cruz), respectively. According to personal communication with the manufacturer, these antibodies target 15–25 amino-acid (aa)-long sequences within the regions aa 1–50 (before FnI_1_) and aa 2,336–2,386 (after FnI_12_) of human Fn (acc.no. P02751, UniProt), respectively. Mouse monoclonal antibody IST2 (1:50, #sc-59825, Santa Cruz) was used to sample FnIII_12-14_ (comprising the heparin II binding site)^63^.

Donkey anti-rabbit IgG (#711-005-152, Jackson) or donkey anti-mouse IgG (#715-005-151, Jackson) were self-labelled with Alexa Fluor 647 succinimidyl ester (#A20006, Invitrogen) or CF680 succinimidyl ester (#SCJ4600055, Sigma-Aldrich) for 1 h at RT, passed through a Zebra microspin desalting column (#89882, Thermo Scientific) to remove unreacted dye, and incubated on samples at concentrations of 40 μg ml^−1^. This high concentration was chosen based on a dilution series in which we confirmed that this concentration of secondary antibodies was not limiting the labelling efficiency. The average degree of labelling for these antibodies was between 1 and 2 dyes per antibody. In addition or alternatively, we used commercial donkey anti-goat IgG Alexa Fluor 647 (1:50, #705-606-147, Jackson) and goat anti-mouse IgG CF680 (1:50, #SAB4600361, Sigma). For the second colour in stepwise photobleaching experiments, a goat anti-mouse Alexa Fluor 488 (1:50, #A11029, Invitrogen) was used.

### Imaging buffers

The imaging buffer for dSTORM contained 200 mM Tris, pH 8.2, 4% w/v glucose, 1 mg ml^−1^ glucose oxidase type VII (#G2133), 0.2 mg ml^−1^ catalase from bovine liver (#C4706), 20 μM tris(2-carboxyethyl)phosphine (TCEP; #646547), 2.5% glycerol and 100 mM cysteamine (#30070, all from Sigma-Aldrich). The cysteamine concentration was chosen higher than in previous work^64^ to increase the dark fraction of dyes. The imaging buffer was mixed from stock solutions shortly before the experiment and stored on ice.

In the imaging buffer for stepwise bleaching, cysteamine was replaced by 2 mM ascorbic acid (AA; #A-0537, TCI) and 2 mM methyl viologen (MV; #856177, Sigma-Aldrich). MV and AA form a redox system that suppresses fluorophore blinking and maximizes photon counts^65^.

### SMLM imaging

In our SMLM setup, the beams of a red diode laser (641 nm, 100 mW; #1150205, Coherent), a blue solid-state laser (488 nm, 20 mW; #FCD488-020, JDSU) and a violet diode laser (405 nm, 100 mW; #90300, Cobolt) were passed through clean-up filters (#LD01–640/8–12.5, Semrock; #ZET488/10x, Chroma; and #LD01–405/10–12.5, Semrock; respectively), combined by two dichroics in series (#FF500-Di01–25x36 and #LM01-427-25, Semrock), expanded by a factor of three, passed through a quarter-wave plate (#AQWP05M-600, Thorlabs) and focused through a wide-field lens (#AC508-300-A, Thorlabs) onto the back-focal plane of a high numerical aperture objective (NA 1.49, TIRF × 60, oil, Olympus). The resulting intensity of the red imaging laser in the focal plane was ∼4 kW cm^−2^ for dSTORM and ∼1.3 kW cm^−2^ for stepwise photobleaching. A focus lock was implemented based on the total internal reflection of a diode laser (#LDM-780-SM7-0.2-P-1-FA, OeMarket) from the coverslip, its detection on a quadrant photodiode (#QPD-PDQ80A, Thorlabs), and a software-based feedback to adjust the objective's z-position by a piezo (Tritor100, Piezosystem Jena). Fluorescence was separated from the excitation by a dichroic mirror (#Di01-R405/488/561/635-25x36, Semrock) and a bandpass filter (ET700/100 for AF647 and CF680, Chroma; or #FF03–525/50-25 for AF488, Semrock) and focused (#AC508-500-A, Thorlabs) onto a back-illuminated EMCCD camera (iXon 897, Andor). For 3D dSTORM by astigmatism, a cylindrical lens (#LJ1516RM-A, Thorlabs) was placed ∼6 cm in front of the camera. For dual colour dSTORM, the field of view was restricted by an adjustable slit placed at an intermediate image plane, emission was split using a longpass imaging dichroic (690dcxr, 5 mm thick, Chroma) placed behind the bandpass filter, and the two emission paths were focused onto separate halves of the CCD sensor. The pixel size in the image was 94 nm as determined by imaging a custom-made normalization grating. The set-up was controlled by home-written LabVIEW software (National Instruments), and the camera was controlled using the open-source software MicroManager (v1.4.15, www.micro-manager.org).

Samples were mounted on a custom holder with a chamber containing 0.2 ml imaging buffer. For dSTORM, the power of the activating UV laser was dynamically adjusted from 0.0001 to 30% during the acquisition to keep the number of localizations per frame approximately constant. Typically 20,000–100,000 frames with 20 or 30 ms exposure time were acquired. For stepwise photobleaching, 1,000 frames with 30 ms exposure time were acquired.

### Data processing for dSTORM

Fitting and analysis of dSTORM movies was performed using home-written software in MATLAB (MathWorks) and was described in detail previously^66^. Camera counts were approximately converted into photon counts using the offset and effective gain of our camera that were determined as described elsewhere^45^. GPU-based maximum likelihood estimation was implemented using open-source code^67^ on a GeForce GTX760 (Nvidia). The calibration for 3D dSTORM was performed by z-stepping of fluorescent beads^66^. Lateral drift of the microscopy stage was in the range of 50–400 nm per hour and corrected for by redundant image correlation based on image features^66^. The residual positional errors were estimated to be less than ∼5 nm. In dual colour dSTORM, an approach similar to ‘spectral demixing'^68^ was used for a robust assignment of the two channels and for their mutual registration^66^. Test experiments with only AF647-labelled or only CF680-labelled antibodies showed that the erroneous assignment of labels to the wrong channel was well below 1% for either case.

A typical histogram of the localization precision for Alexa Fluor 647 peaked around ∼6 nm, while that for CF680 peaked around ∼8 nm. The CF680 dye was chosen over Alexa Fluor 700 because it showed superior spectral and photophysical properties for dual colour dSTORM in our experiments.

For visual representation, localizations within 100 nm and spanning subsequent frames were combined into a single localization and then filtered according to their z-position (−1…+1 μm) and localization precision (<12 nm). Localizations were colour-coded according to their z-position (or channel) and rendered according to their fitted localization precision and intensity.

All images are depicted as a Gaussian rendering of the localizations.

### Data processing for stepwise photobleaching

Fitting and analysis of stepwise photobleaching movies was done with home-written software in MATLAB (MathWorks). From the fifth image in the movie, isolated intensity maxima above a user-defined threshold were chosen as centres for regions of interest (ROIs) with a size of 9 × 9 pixels. Spots whose ROIs contained overexposed pixels in any frame or that did not bleach completely were excluded from further analysis. Intensity-time traces were extracted for each ROI from the photon-count converted movie (see above). Intensity plateaus in the time trace (with a minimum length of five frames) and the optimum number of steps were identified by a step-finding routine^69^. Time traces for which the found step heights varied by more than a factor of two, showed upward steps, or did not properly resemble the data were excluded from further analysis. For each spot, ROI frames within plateaus were summed up. For each step, the summed ROI after a step was rescaled to the length of the plateau before the step and subtracted from the respective summed ROI. The difference ROIs were least-square fitted using a two-dimensional Gaussian with the x and y position, the width of the Gaussian, the intensity, and a constant background as free fitting parameters. When the residual of a fit showed systematic deviations from homogeneous noise, the whole spot/timetrace was discarded.

The localization precision in stepwise photobleaching experiments was determined according to the Cramèr-Rao lower bound as recently described^70^. For all localizations, the precision was better than 2 nm; the error of the distance between two localizations was accordingly smaller than ∼3 nm.

### Measurement of end-to-end distances

The end-to-end distance of Fn dimers in thin fibrils were measured from Fn matrix containing sparsely incorporated Fn-AF647^N-terminal^ dimers.

For dual colour dSTORM, a CF680 co-staining was used to identify protofibrils and to perform the drift correction. Signal spots in the AF647 channel that lay within fibrils were manually selected and a line was drawn along the direction of the fibril (Fig. 2c). The line profile in the AF647 channel showed two prominent peaks and was fitted by the sum of two Gaussians to obtain the distance between N-terminal labels, and therefore, the end-to-end distance.

For stepwise photobleaching, an AF488 co-staining was used to acquire a separate epifluorescence image of the Fn matrix. This image was registered with respect to the first image of the stepwise photobleaching movie by image cross-correlation. The fitted dye positions of spots that showed two bleaching steps were overlaid on the composite image (see Fig. 2g). Molecules that localized to the centre of thin, isolated Fn fibrils were manually selected, and the respective distances were pooled.

### Periodicity analysis of label patterns

For analysing the arrangement of Fn molecules in Fn fibrils, fibril sections of 0.5–1 μm in length were selected by manually drawing a line. Localizations within 100 nm perpendicular to this line were rendered as described above to a pixel size of 2.5 nm; their z-position was neglected (=z-projection). Intensity profiles along fibrils were generated by a perpendicular plot profile (ImageJ).

The labelling periodicity was assessed by an autocorrelation analysis. Line profiles of fibril sections were considered to be periodic if the autocorrelation contained four or more maxima at regular distances. To prevent a biasing of this manual selection towards specific periodicities, the *x* axis of the line profile and of the autocorrelation was left unlabelled. The manual selection was tested with four independent people from our laboratory on one data set (C20 antibody labelling of thin Fn fibrils). The rejection rate for each person was between 30 and 40%. Thirty-five percent of the data set was chosen by all users. The agreement between two users was >80%. Moreover, the unbiasedness and precision of the analysis was tested on simulated data (Supplementary Fig. 4b). The dominant periodicity was determined from the first maximum in the autocorrelation. The position of the maximum was refined by a quadratic interpolation using the neighbouring data points before and after the maximum (see Fig. 3d).

For dual colour images, line profiles were generated from fibril sections as described above. To assess the relative positions of different labels, a normalized cross-correlation was calculated from the line profiles of the two channels. All calculations were done in Mathematica 8.0 (Wolfram Research).

### Analysis of Fn fibril thickness and bundling

About 200 nm long and 50 nm wide sections along fibrils were analysed with an image analysis script written in MATLAB (MathWorks). Localizations were projected perpendicular to the fibril axis, binned into 2 nm bins and fitted by a Gaussian with a constant offset.

The fibril diameter was defined as the full width at half-maximum (FWHM) of the Gaussian fit according to the relation FWHM ∼2.3 σ. It has to be noted that this definition comprises contributions from localization imprecision and labelling inaccuracy. It thus only provides an upper limit for the true fibril diameter.

The localization density was calculated by dividing the number of localizations within the analysed box by the box length after subtracting background localizations that are represented by the constant offset of the fit. The localization density serves as an approximate measure for the amount of Fn within the fibril. We therefore assumed that the accessibility of binding sites is equal in fibrils of different thickness. The stochastic nature of labelling and dye blinking led to an inherent scattering of the observed number of localizations for even the very same fibril thickness. To increase the reliability of measurements, we averaged three measurements along the same fibril. To eliminate the variability between different samples, measurements and different branching levels, only relative differences between regions before (*B*) and after (*A*) a branching point were analysed. To this end, we normalized the difference of the localization density from before and after the branching point to the density of the bundled fibril. This relative difference (*Δ*) was calculated according to *Δ*=(*B*-*A*1-*A*2)/*B*. dSTORM images from lysine-labelled Fn assembled into the matrix (5% fraction of lysine-labelled Fn conjugates of exogenous added Fn) and immunolabelled fibrils (poly-clonal ab23750) yielded consistent results and and were pooled for the junction analysis.

More details about the used procedures and a validation of the analysis procedure by Monte Carlo simulations can be found in the Supplementary Note. The computer code for fibril analysis can be requested from the authors.

## Additional information

**How to cite this article:** Früh, S. M. *et al*. Molecular architecture of native fibronectin fibrils. *Nat. Commun.* 6:7275 doi: 10.1038/ncomms8275 (2015).

## Supplementary Material

Supplementary Information Supplementary Figures 1-9, Supplementary Note 1 and Supplementary References
