# Supplementary material for: Molecular architecture of native fibronectin fibrils
Source: Nat Commun. 2015 Jun 4;6:7275. doi: 10.1038/ncomms8275 (PMC4468872; doi:10.1038/ncomms8275)
Supplement: Supplementary Information — Supplementary Figures 1-9, Supplementary Note 1 and Supplementary References [file ncomms8275-s1.pdf]

Supplementary Information for the article

Molecular architecture of native fibronectin fibrils

Susanna Maria Früh*, Ingmar Schoen*, Jonas Ries, Viola Vogel

* contributed equally

Correspondence: V. V. (viola.vogel@hest.ethz.ch) , I. S. (ingmar.schoen@hest.ethz.ch)

Content:

Supplementary Figures .... p. 2-10

Supplementary note: stochastic simulations and analysis of dSTORM data.. p. 11-14

Supplementary references ………………………………………………………… p. 15

Supplementary Figures

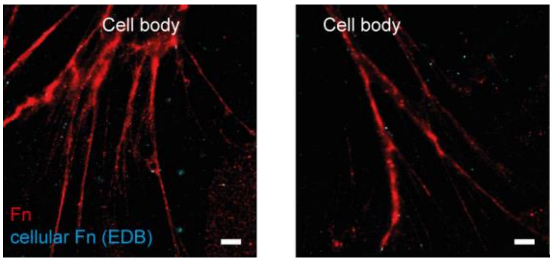

Supplementary Figure 1: **Qualitative estimation of matrix composition regarding content of cellular and plasma Fn.**
Dual color dSTORM images of Fn ECM. Cellular Fn is stained with anti-EDB (L19, blue) and the contour of the matrix is stained with a polyclonal anti-Fn (ab23750, red). The blue channel is pictured in an overexposed way for better visualization of the sparse EDB labels. Cellular Fn was rarely observed. Scale bars 1 µm.

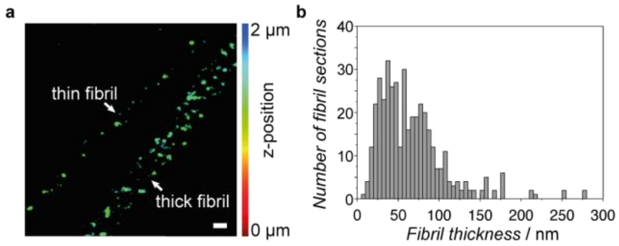

Supplementary Figure 2: **Fibril thicknesses in Fn ECM.**
a) 3D dSTORM image of IST2-AF647 labeled fibrils. Scale bar 100 nm.
b) Histogram of Fn fibril thickness (n=409, from 5 independent cells). Thickness was defined as the full-width at half-maximum (FWHM).

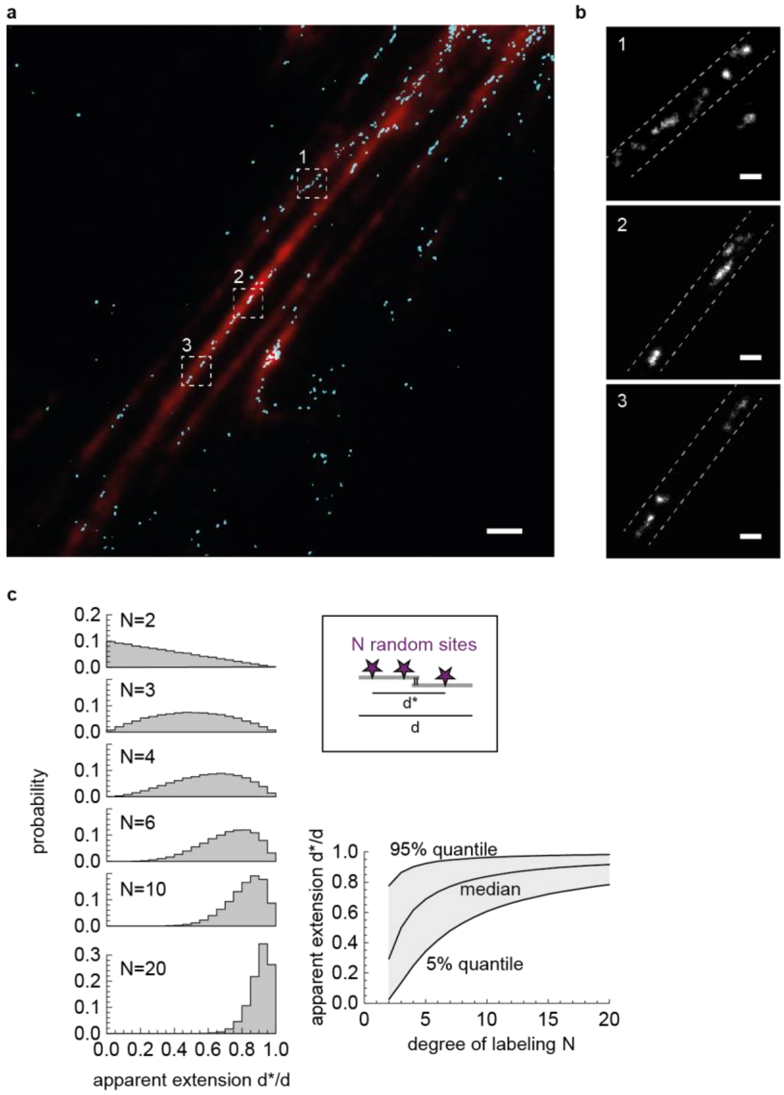

Supplementary Figure 3: **Extension of random labeled single Fn molecules in protofibrils.**
Lysine-labeled Fn-AF647 conjugates (degree of labeling = 22) were incorporated into the Fn ECM at high dilution for measuring the contour length of single molecules.
a) dSTORM image with Fn-AF647 (blue) is overlayed with a brightfield image of IST2-AF488 (red). The blue channel is pictured in an overexposed way for better visualization of the sparse Fn-AF647 labels. Scale bar 1 µm.
b) Magnification of areas from a) with examples showing the difficulties of measuring the contour length. No clear assignment to one molecule was possible due to partly interrupted punctate label patterns. Scale bars 100 nm.
c) Theoretical analysis of contour length measurements assuming random lysine labeling. Box: N labels are randomly distributed along the Fn dimer with length d. The apparent contour length d* is the distance between the outermost labels. Left: Probability distributions for d* for different degrees of labeling. Lower Right: Dependence of the apparent contour length on the number of labels. Due to the unlabeled ends, the apparent contour length severely underestimates the true extension of the molecule.

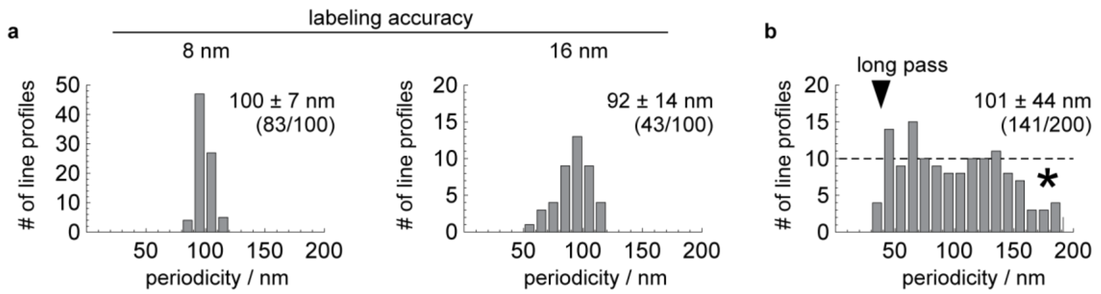

Supplementary Figure 4. **Analysis of simulated line profiles.**
See supplementary note for details of the simulations. The exactly same procedure was used as for experimental data.
a) Effect of the labeling accuracy. The same parameters as in Supplementary Fig. 9c were used.
b) Test for biasedness. 200 line profiles were generated with a periodicity that was picked uniformly from the interval 10…200 nm. Labeling accuracy was 8 nm, labeling efficiency 0.9. Arrowhead: cutoff due to localization precision. Asterisk: rejection due to selection criterion.

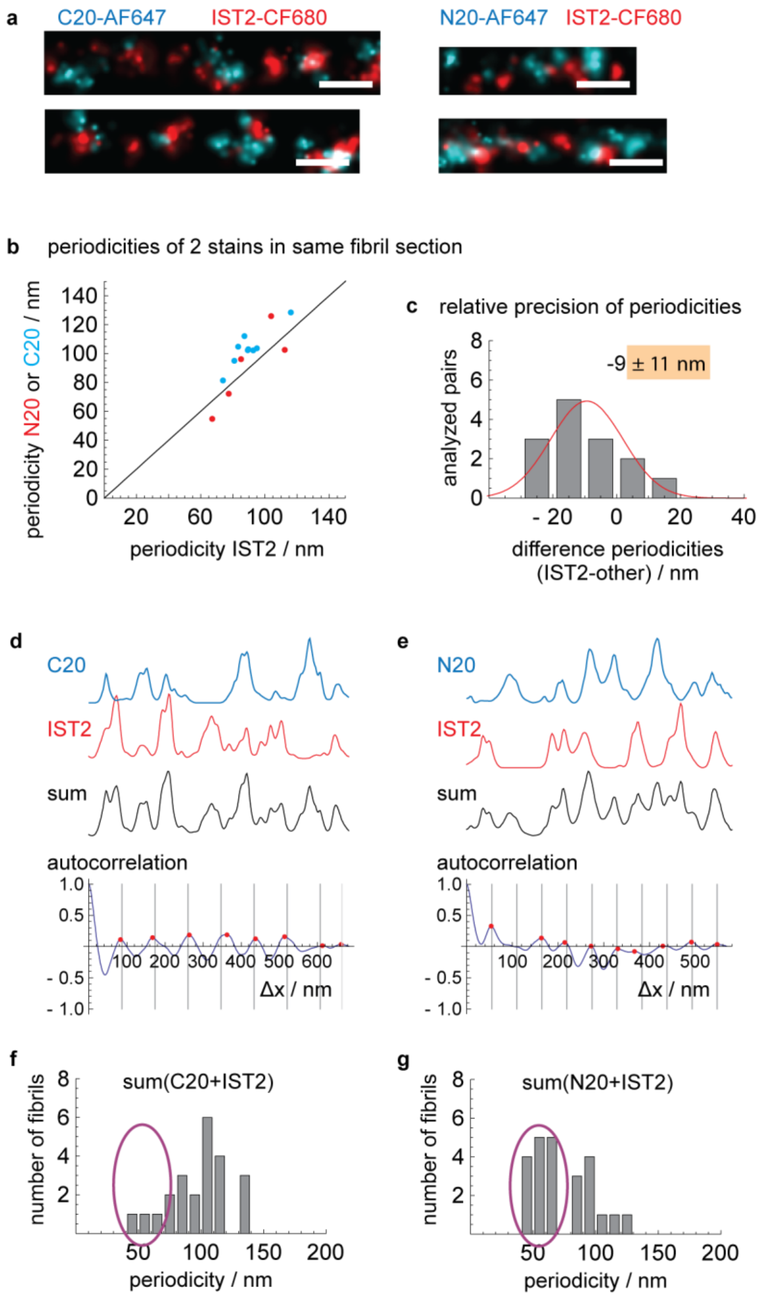

Supplementary Figure 5: **Analysis of dual color labeling patterns of different antibodies.**
a) Examples for dual color dSTORM images: Immunofluorescence fibril labeling of Fn with C20-AF647/IST2-CF680 and N20-AF647/IST2-CF680. Scale bars 100 nm.
b) Scatter plot of the periodicities obtained from different labels for the same fibril section. Line: case of perfect agreement.
c) Differences between periodicities obtained from different labels for the same fibril section. The width of the distribution was 11 nm and can be taken as a measure of the precision with which the periodicity was determined. Due to the worse quality of dual color dSTORM images compared to single color dSTORM images, this estimate is an upper limit for the measurement error in Fig. 3.
d-g) Apparent periodicity of summed dual color images as a model for staggered fibrils.
d+f) C20 and IST2 labels were mainly in phase and resulted in similar periodicities as the individual labels.
e+g) N20 and IST2 labels were interlaced and resulted in line profiles with an apparently doubled frequency / halved periodicity (highlighted in g).

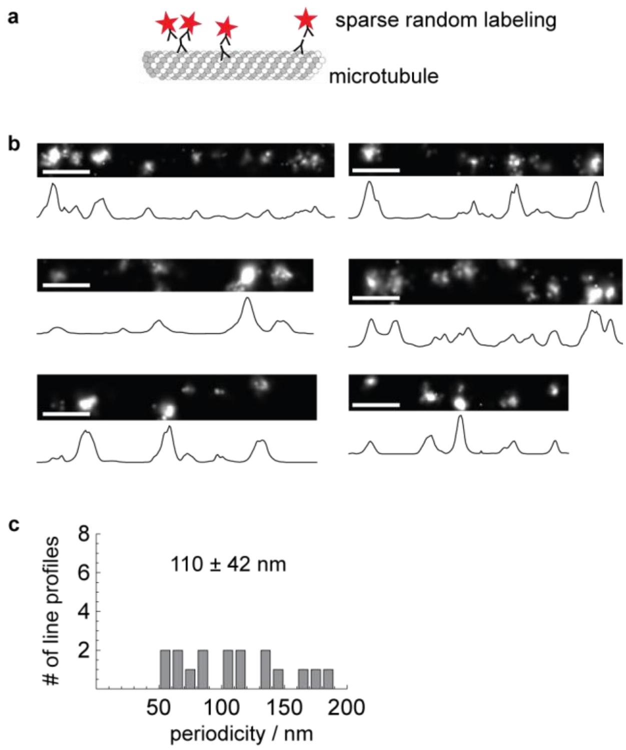

Supplementary Figure 6: **False positive control with a random distribution of labels.**
a) Microtubules were polymerized from rhodamine-labeled tubulin (Cytoskeleton), adsorbed to a polylysine-coated coverslip, and immunolabeled (anti-α tubulin, ab7750, Abcam; secondary with AF647). The antibody concentration was chosen so low (dilution 1:200) that the labeling along microtubules was discontinuous. The stochastic sampling of binding sites along the microtubule at this labeling density resulted in a random labeling.
b) Examples for dSTORM images of immunolabeled sections along microtubules with the respective intensity line profiles. Scale bars 100 nm.
c) Result of the periodicity analysis. 58 % of the n=40 line profiles were rejected (for comparison: 30-40 % of Fn line profiles were typically rejected). The ‘fake’ periodicities of the cases that showed a periodic autocorrelation ranged from 60 to 190 nm. No preferred frequency was visible, as expected for a random arrangement of labels. Numbers are given as mean +/- s.d..

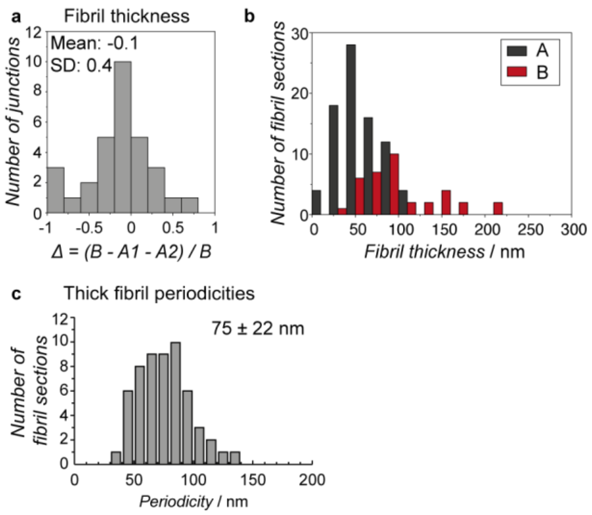

Supplementary Figure 7: **Characterization of bundling to thick fibrils in the cell periphery.**
a) Histogram of the relative difference in fibril thickness of fibrils before and after a junction (see Fig. 5c for definition). Fibril thickness was determined from the full-width at half-maximum (FWHM) of a Gaussian fit of a perpendicular line profile (n=31, from 5 independent cells).
b) Histogram of fibril thicknesses color-coded according to their relative position to a junction (black: after; red: before; see Fig. 5c for definition; n=117 fibrils, from 5 independent cells).
c) Histogram of labeling periodicity of thick fibrils. Compared to the histogram for thin fibrils (Fig. 3c), an additional, pronounced shoulder below 70 nm was observed (n=56, from 5 different cells). Numbers are given as mean +/- s.d..

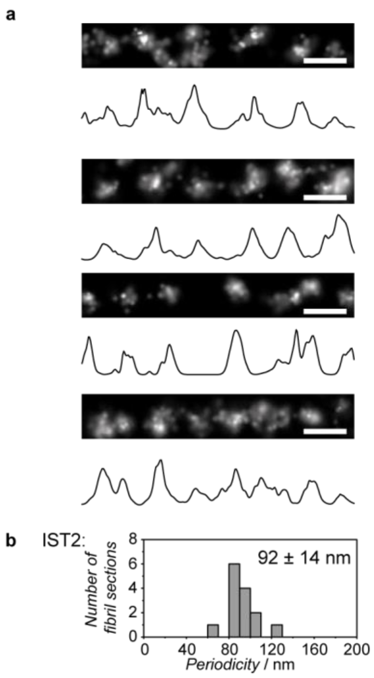

Supplementary Figure 8: **Periodicity of labeling patterns in cellular Fn matrix.**
Cells were cultured in Fn-depleted medium, resulting in a Fn matrix mainly composed of cellular Fn.
a) Mapping of epitopes by immunolabeling using antibody IST2. Shown are dSTORM images and respective line profiles for 4 representative fibrils sections. Scale bars 100 nm.
b) Histogram for the periodicity of IST2 staining (n=14, from 5 independent cells). Numbers are given as mean +/- s.d.

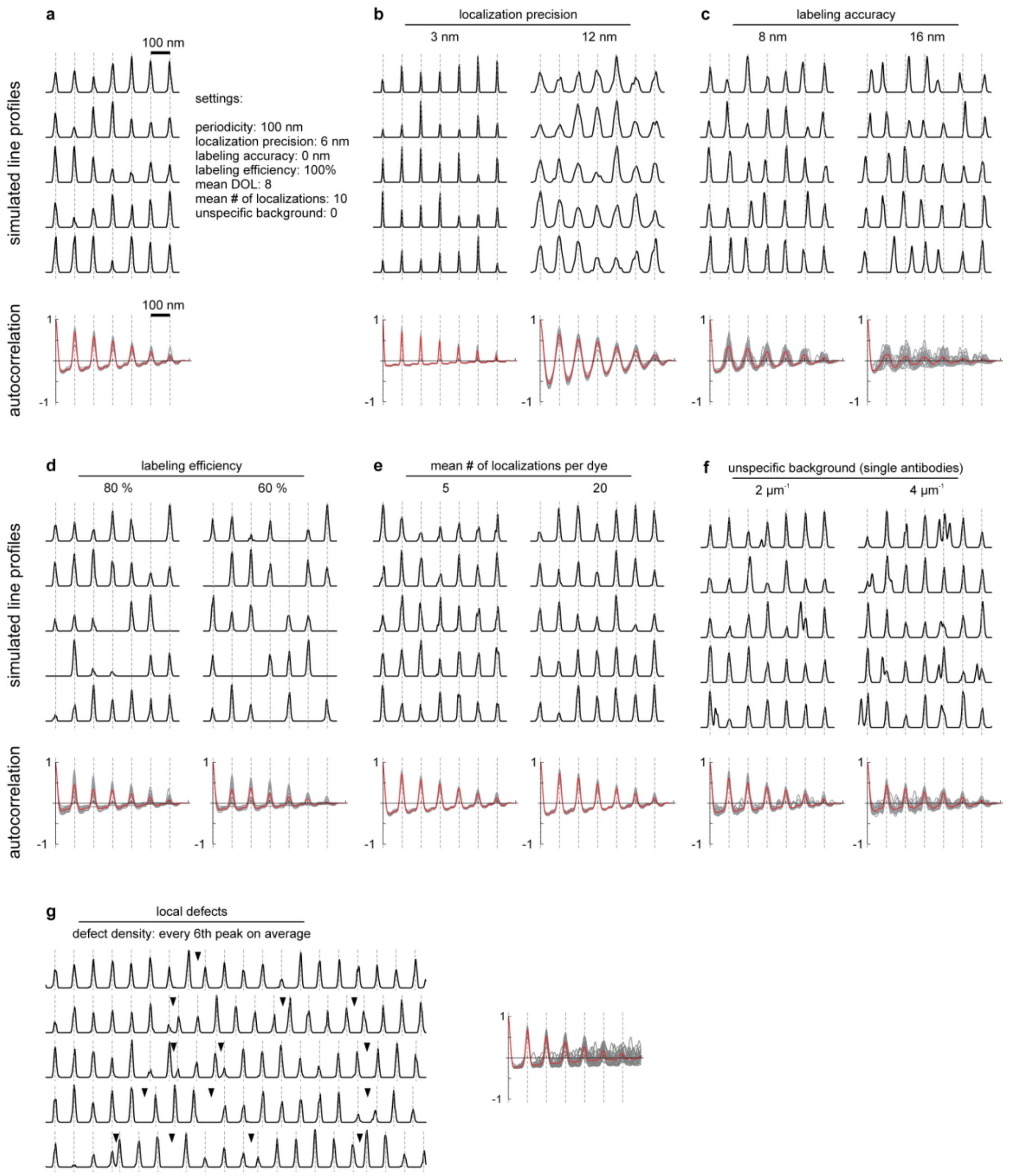

Supplementary Figure 9. **Influence of labeling and localization parameters on simulated line profiles**.
Shown are 5 line profiles per condition (top), as well as 20 autocorrelations (gray) and their average (red, bottom).
a) Reference settings. The other subpanels show the effect of variations of a single parameter, namely
b) the localization precision,
c) labeling accuracy,
d) labeling efficiency,
e) mean number of localizations per fluorophore,
f) background from unspecific antibody binding,
g) local defects (arrow heads) of shorter/longer segments.

Supplementary note

The purpose of this section is to explain the stochastic nature of the dSTORM data and what consequences arise for the analysis of fibril periodicity. Most experienced users of dSTORM will already be aware of these issues; we repeat them here because they are also the foundation of stochastic simulations that can be used to elucidate which parameters are crucial for the periodicity analysis. We show the benefits of performing an autocorrelation analysis, and test its limitations with simulated data.

Stochastic sampling of linear periodic structures

The analysis of periodic biological structures is complicated by non-deterministic factors arising from the labeling procedure (points 1-3), the imaging by localization microscopy (4-6), and irregularities present in the sample itself (7-8).

1) Labeling efficiency.
   The labeling efficiency is given as the fraction of labeled binding sites, e.g. as p_0 = 90 %. When the regularly arranged binding sites for (primary and secondary) antibodies are not completely saturated, some sites will not carry a label and thus do not give rise to a signal. These void sites will appear as irregularities in the observed label pattern. The probability that a certain binding site is labeled is given by the binomial distribution with 1 trial and success rate p_0.

2) Unspecific labeling.
   The quality of immunofluorescence stainings depends on the affinity and selectivity of the used antibodies. Unspecific binding along the investigated fibril leads to additional peaks in the intensity line profile at ~random positions. There is no intrinsic criterium that distinguishes between these false peaks and the peaks at the specific binding sites.

3) Labeling accuracy.
   Due to the finite size of antibodies, the fluorophores are not exactly positioned at the binding site on the investigated structure but at a certain distance in a ~random direction. This distance between label and binding site has been estimated from STORM/PALM images of immunolabeled microtubules and is on the order of σ_{Ab} ~5…10 nm.[1] When the orientation of the antibody sandwich is random, the probability of finding an antibody at position x_{Ab} when the position of the binding site was at x_0 is approximately described by a normal distribution according to equation (0).

$$p\left(x_{Ab} \mid x_0, \sigma_{Ab}\right) = \left(1/2\pi\sigma_{Ab}^2\right)^{1/2} \exp\left(-\left(x_{Ab} - x_0\right)^2 / 2\sigma_{Ab}^2\right) \qquad (0)$$

   This labeling inaccuracy causes a lateral deviation of the peak positions in the intensity line profile from their true positions, leading to irregularities in the

spacing between peaks.

4) Number of fluorophores per antibody.
Antibodies are typically labeled using NHS-chemistry. Commercially available labeled secondary antibodies have an *average* degree of labeling (DOL) of DOL ~5…10. This average DOL can e.g. be determined from absorption measurements. Due to the large number of lysines in proteins and i.e. in antibodies, the labeling is stochastic and the exact number of fluorophores varies from antibody to antibody. The number of labels follows from a binomial distribution that describes the probability to get k labels on n lysines with success rate p for each lysine. With the average $DOL = n \cdot p$ and in the limit $n \rightarrow \infty$ the binomial distribution results in a Poisson distribution according to equation (0). It describes the probability that an antibody with a certain average DOL carries k fluorophores.

$$p\left(k \mid DOL\right) = \frac{e^{-DOL} DOL^{k}}{k!} \qquad (0)$$

The number of localizations will roughly be proportional to the number of fluorophores on an antibody. Hence, the variation in the number of dyes mainly affects the height of peaks in the intensity line profiles. Since the probability for an antibody carrying no fluorophore at all is not zero, these non-labeled antibodies contribute to non-labeled sites of the periodic structure, equivalently to a non-perfect labeling efficiency.

5) Number of localizations per fluorophore.
dSTORM takes advantage of the on/off blinking of fluorophores. For AlexaFluor647 in a buffer containing 100 mM cysteamine, the blinking is caused by redox reactions and therefore stochastic. The number of "on"-events before irreversible bleaching roughly follows a Poisson distribution with mean λ according to equation (0).

$$p\left(k \mid \lambda\right) = \frac{e^{-\lambda} \lambda^{k}}{k!} \qquad (0)$$

The average number of localizations per dye under our experimental conditions was ~8-15. The variation in the number of localizations mainly affects the height of peaks in the intensity line profiles.

6) Localization precision.
The localization precision is fundamentally limited by the amount of collected photons and by other factors such as background fluorescence, camera noise, or (incompletely corrected) stage drift. The position of localizations will thus scatter around the true position. For a certain fixed precision σ_{dye}, the probability of localizing the fluorophore at position x when its true position was μ_{dye} follows a normal distribution according to equation (0).

$$p\left(x \mid x_{Ab}, \sigma_{dye}\right) = \left(1/2\pi\sigma_{dye}^{2}\right)^{1/2} \exp\left(-\left(x - x_{Ab}\right)^{2} / 2\sigma_{dye}^{2}\right) \qquad (0)$$

The finite localization precision leads to a broadening of peaks in the intensity line profiles. It thus has the effect of a low-pass filter and sets a limit for the resolution of periodicities with periods shorter than $< 2\sigma_{dye}$.

7) Fibril elasticity.
Proteins have been shown to behave as elastic materials under small forces, ranging from unstructured polymers acting as entropic springs, over deformation of tertiary structure, to (ir)reversible unfolding of domains. Assuming homogeneous material properties along fibrils such that each repeating unit experiences the same deformation, elastic stretch or relaxation can lead to variations in the actual periodicity. For example, Fn fibril strains have been shown to vary by a factor of 4 in the ECM.[2]

8) Local defects.
Fibrils assembled from regular building blocks will be periodic, but the incorporation of a longer/shorter building block leads to a perturbation of the ordered arrangement. In the case of Fn, such local defects could arise from splice variants of different lengths due to extra domains A/B or the variable regions[3], local spontaneous or force-induced unfolding of tertiary structure[4], or alternative Fn-Fn interactions between adjacent molecules. As a result, fibrils will be periodic on shorter length scales but do not possess a long-range order.

Computer simulations

A model was constructed based on the deterministic features of the periodic structure and the stochastic behavior of components was simulated using Monte Carlo sampling of the appropriate probability distributions. The following procedure was used: positions of antibodies were drawn from the periodic grid along a fibril, deviations from labeling inaccuracy were added, and non-labeled sites were removed. The number of localizations per antibody was drawn from the product of Poisson distributions for the DOL and for the number of localizations. The positions of these localizations were generated from the true antibody position and the localization precision. Background dyes/antibodies were added at random positions and localizations were generated as before. In analogy to the reconstruction of experimental dSTORM images, the x positions of all generated localizations were binned into 2.5 nm bins and smoothened by a Gaussian low-pass filter with a width $s = 0.4 \cdot \sigma_{dye}$.

The simulation results illustrate the effect of changes in the discussed parameters (Supplementary Fig. 9). Incomplete (Supplementary Fig. 9d), inaccurate (Supplementary Fig. 9c), or unspecific labeling (Supplementary Fig. 9f), as well as local defects (Supplementary Fig. 9g) change the distance between neighboring peaks and therefore pose the main challenge for the analysis. The other parameters merely lead to variations in peak intensities and minor variations in the position of maxima.

Autocorrelation analysis of stochastic labeling patterns

A nearest neighbor analysis of peaks in the line profiles is the most direct way to analyze the periodicity. However, nearest neighbor distances are affected by all of the stochastic parameters described above.

To overcome at least some of these shortcomings, we base our analysis on the spatial autocorrelation of the line profile. More precisely, we analyze the position of the first peak in the autocorrelation, which essentially is the average over the most prominent nearest neighbor distances within a single line profile.

Supplementary figure 8 also shows how the autocorrelation is affected by the stochastic parameters. The autocorrelation is intrinsically robust against stochastic variations in the number of localizations (Supplementary Fig. 9e) and the localization precision (Supplementary Fig. 9b). It also overcomes the undersampling problem due to incomplete labeling (Supplementary Fig. 9d). Local defects mainly affect the higher order peaks in the autocorrelation but barely the first peak (Supplementary Fig. 9g).

However, the autocorrelation was sensitive to the labeling accuracy and showed significant variation between simulated line profiles, i.e. with increasing inaccuracy (Supplementary Fig. 9c). It is intuitively clear that this parameter is the most critical one when it comes to the task of distinguishing periodic (but disturbed) line profiles from a random arrangement of labels. To quantify the precision of the analysis procedure, we analyzed n=100 simulated line profiles with a nominal periodicity of 100 nm but different labeling accuracy. With increasing inaccuracy, both the fraction of rejected lines profiles as well as the width of the distribution of found periodicities increased (Supplementary Fig. 4a). The measurement precision was affected nearly 1:1 by the labeling accuracy; for the labeling accuracy of immunofluorescence σ_{Ab} ~ 8 nm, the precision was ~7 nm. This precision roughly agreed with the experimentally determined precision of 11 nm (from dual color dSTORM, see Supplementary Fig. 5c). The rejected fraction of simulated line profiles (17 %) was smaller than when analyzing Fn fibrils (30-40%), probably because the periodicity in the simulations was not compromised by the presence of two labeling sites per Fn dimer (see Fig. 3a in the main text), or by non-specific background and/or local defects.

To test whether the selection criterion or the peak detection were biased and which frequencies could be detected, lines profiles with variable periodicity, covering uniformly the range between 10…200 nm were generated and analyzed. Supplementary figure 4b shows that the periodicities were correctly retrieved over the range from 50…150 nm. At smaller periodicities than ~40 nm, the localization precision acts as a long-pass filter (arrowhead). At large periodicities (asterisk), the line profile/autocorrelation was discarded either due to an insufficient number of peaks or due to an erroneous identification of a small hump as first peak.

In summary, the autocorrelation analysis procedure can be expected to yield valid results for periodicities between ~40…170 nm with a precision better than 10 nm.

Supplementary references

1. Ries, J., Kaplan, C., Platonova, E., Eghlidi, H. & Ewers, H. A simple, versatile method for GFP-based super-resolution microscopy via nanobodies. *Nat. Methods* **9,** 582–584 (2012).
2. Ohashi, T., Kiehart, D. P. & Erickson, H. P. Dynamics and elasticity of the fibronectin matrix in living cell culture visualized by fibronectin-green fluorescent protein. *P. Natl. Acad. Sci. Usa* **96,** 2153–2158 (1999).
3. Schwarzbauer, J. E. & DeSimone, D. W. Fibronectins, their fibrillogenesis, and in vivo functions. *Cold Spring Harb Perspect Biol* **3,** :a005041 (2011).
4. Erickson, H. P. Reversible unfolding of fibronectin type III and immunoglobulin domains provides the structural basis for stretch and elasticity of titin and fibronectin. *P. Natl. Acad. Sci. Usa* **91,** 10114–10118 (1994).
